# Formation of PEG-PLGA Microspheres for Controlled Release of Simvastatin and Carvacrol: Enhanced Lipid-Lowering Efficacy and Improved Patient Compliance in Hyperlipidemia Therapy

**DOI:** 10.3390/polym17050574

**Published:** 2025-02-21

**Authors:** Lin Fu, Hengxin Ren, Chaoxing Wang, Yaxin Zhao, Bohang Zou, Xiangyu Zhang

**Affiliations:** 1 College of Pharmacy, Jiamusi University, Jiamusi 154007, China; fulin@jmsu.edu.cn (L.F.); renhengxinjmsu@outlook.com (H.R.); wangchaoxing99@outlook.com (C.W.); xiaozou9898@outlook.com (B.Z.); 2 College of Heilongjiang, University of Chinese Medicine, Jiamusi 154007, China; zyxfsci@outlook.com

**Keywords:** simvastatin, carvacrol, microsphere, pharmacokinetic, hypolipidemia

## Abstract

Polymer-based drug-controlled release systems offer greater efficacy and potency than conventional therapies. However, prominent drug side effects, lower circulation, and low drug loading capabilities limit their application range. In this work, the combination of Simvastatin (SIV) and Carvacrol (CAV) into PEG-PLGA microspheres (SIV-CAV-PP-MS) was achieved via an emulsification-solvent evaporation technique, resulting in microspheres characterized by high encapsulation efficiency and reduced particle size. In vitro studies demonstrated that the cumulative drug release increased with higher SIV and CAV levels in the release medium, reaching 88.91% and 89.35% at 25 days. Pharmacokinetic analysis revealed that the concentrations of SIV and CAV reached their maximum levels at approximately seven days in the SIV-CAV-PP-MS group, which indicates that using PEG-PLGA as a carrier significantly delays drug release. In vivo, evaluation demonstrated that the SIV-CAV-PP-MS high-dose group and positive drug control group showed reductions in low-density lipoprotein cholesterol levels by 0.39-fold and 0.36-fold compared to the Hyperlipidemia model group, and the addition of CAV significantly enhanced the lipid-lowering effects of SIV. Histological examinations indicated that the SIV-CAV-PP-MS medium-dose group displayed histological features more closely resembling those of normal mice compared to the Simvastatin control group, with a well-organized hepatocyte structure, a significant reduction in lipids, and improved liver health. The prepared polymeric microsphere utilizing SIV and SAV will be a promising dosage form for hyperlipidemia disease patients, with superior lipid-lowering efficacy and improved patient compliance.

## 1. Introduction

Hyperlipidemia is a prevalent lipid and lipoprotein metabolism disorder and is a significant risk factor for various cardiovascular and cerebrovascular diseases [1]. Statin families, particularly simvastatin (SIV), due to their anti-hyperlipidemic effect through lowering the low-density lipoprotein, are one of the most recommended therapies for the primary and secondary prevention of cardiovascular disease [2,3]. However, lipid-lowering medications are associated with a wide range of side effects, such as myotoxicity and hepatotoxicity [4]. Consequently, many patients with hyperlipidemia discontinue SIV therapy due to the severity of these side effects [5,6]. To address the side effects associated with SIV, Ahmed U Ali et al. found that Microsponge SIV preparation was safer than free SIV in minimizing myotoxicity, and SIV microsponges significantly decreased the myotoxicity of SIV [7]. Bing Jiang et al. showed that astragaloside reversed SIV-induced skeletal muscle injury. Astragaloside can be used as a potential drug to reduce the side effects of statins [8]. Currently, research conducted both domestically and internationally is limited to verifying the therapeutic effects of drugs on the side effects of SIV. Hence, there is an urgent need to investigate drug combinations that can mitigate medication-induced side effects while effectively treating hyperlipidemia.

The combined therapy of statins with other kinds of lipid-altering drug is a good choice in clinical practice [9,10]. Carvacrol (CAV), a plant-derived compound known for its potent antioxidant properties, is a co-therapy [11]. Carvacrol demonstrates promising potential in ameliorating the dysfunction of hepatic and endothelial cells in vitro [12]. Khalil M et al. have reported that CAV counteracted lipid accumulation and oxidative stress in hepatocytes and protected endothelial cells from oxidative stress and dysfunction [12]. Soomin Cho et al. revealed the mechanism of CAV inhibition of visceral fat and pro-inflammatory cytokine production. These modes of action in modulating genes associated with adipogenesis and inflammation by CAV are considered relatively novel [13]. When administered alongside lipid-lowering agents, CAV can function as a modulator. It is affirmed that CAV can reduce the side effects caused by Simvastatin by alleviating oxidative stress and addressing Coenzyme Q10 deficiency [14]. Hence, combining both SIV and CAV into a single formulation will enhance the lipid-lowering effect, reduce the dosage of statins, and decrease the risk of adverse effects.

Simvastatin, a lipophilic drug, has low bioavailability and tends to accumulate in adipose tissue, leading to adverse reactions [15]. Traditional carriers, however, cannot mitigate this issue, as they are rapidly cleared by the reticuloendothelial system within seconds to minutes after intravenous injection [16]. Developing new devices designed with medication stability and sustained-release capabilities as drug delivery systems is crucial in biomedicine. Polymer-based drug-controlled release systems offer greater efficacy and potency than conventional therapies, leading to fewer side effects [17,18]. Jusu, S.M. et al. encapsulated targeted drugs using a single solvent evaporation technique with a blend of FDA-approved poly lactic-co-glycolic acid-polyethylene glycol (PLGA-PEG) polymer microspheres. The results show that the blended microcapsules enable the extended release of cancer drugs over 62 days, which could greatly facilitate localized treatment [18]. PEG-PLGA as a carrier material has been proven to reduce macrophage uptake [19]. Subsequently, many studies have demonstrated the prolonged circulation of PEG-PLGA particles in mice [20].

In this study, a SIV-CAV-loaded long-circulating microsphere drug delivery system was developed using amphiphilic PEG-PLGA copolymers. This system aims to leverage the advantages of PEG-PLGA as a carrier to improve the encapsulation efficiency of lipophilic drugs, allow for stable and controlled release of the drug in vivo, extend its half-life, reduce accumulation in non-target tissues, and enhance its bioavailability. Furthermore, the SIV-CAV-loaded microspheres (SIV-CAV-PP-MS) were meticulously characterized using scanning electron microscopy (SEM), laser particle size analysis, differential scanning calorimetry (DSC), and Fourier transform infrared spectroscopy (FTIR). An external drug release experiment was conducted to elucidate the mechanism underlying the microspheres’ drug release profile, and the impact of accelerated release was rigorously examined. Additionally, pharmacokinetic assessments were performed comparing SIV-CAV-PP-MS with SIV-CAV suspension in rats. The study further explored the protective potential of SIV-CAV-PP-MS against statin-induced liver and skeletal muscle injury in hyperlipidemia mouse models.

## 2. Materials and Methods

### 2.1. Materials

Simvastatin, carvacrol, alginic acid, lovastatin, and methyl eugenol were procured from Shanghai Aladdin Biochemical Technology Co., Ltd. (Shanghai, China). PEG (Mn = 2000 g/mol) and PLGA (Mn = 28 kDa) were obtained from the Shandong Academy of Pharmaceutical Sciences (Jinan, China). Polyvinyl alcohol was purchased from Qingdao Yousuxo Chemical Technology Co., Ltd. (Qingdao, China). Methylene chloride, acetidine, acetonitrile, methanol, and formic acid were acquired from Tianjin Kemio Chemical Reagent Co., Ltd. (Tianjin, China). All other chemicals and solvents used in this study were of analytical grade.

### 2.2. Preparation of SIV-CAV-Loaded MS (SIV-CAV-PP-MS)

**Figure 1:**
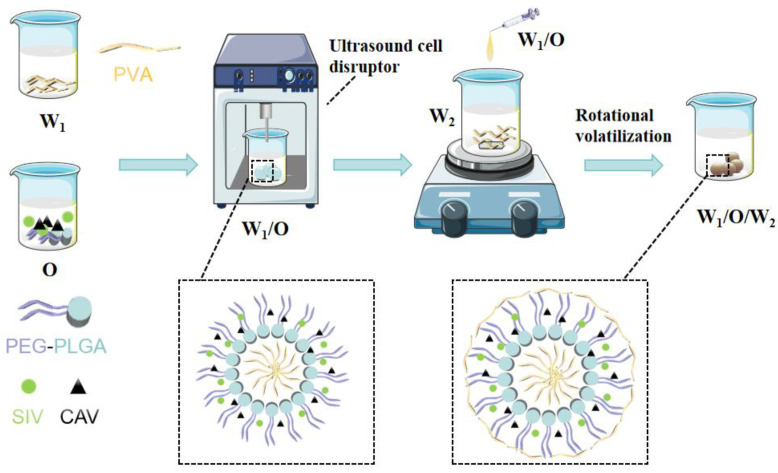
Schematic diagram of SIV-CAV-PP-MS preparation.

The water-oil-water (W_1_/O/W_2_) emulsification-solvent evaporation method is a straightforward and effective method for preparing stable microspheres [21]. This method was employed to prepare SIV-CAV-PP-MS (Figure 1).

First, a 1% polyvinyl alcohol (PVA) aqueous solution (inner water phase W_1_) was emulsified with a drug molecule and a PEG-PLGA solution (polymer organic phase) to form W_1_/O droplets. The formulation was then subjected to probe sonication (JY92-2D, Ningbo Xinzhi Biotechnology Co., Ltd., Ningbo, China) with 30% ultrasonic power for 5 min (utilizing a cycle of 1-s on and 1-s off) at 0 ± 1 °C, using ultrasonic waves to disrupt the particles. The resulting dispersion was slowly added as a thin stream into a continuous phase of 5% PVA (20 mL) while stirring at 1500 rpm using a magnetic stirrer to form a stable emulsion. Stirring was continued for 5 h to allow the evaporation of the organic solvent, leading to the formation of solid spherical microspheres. The supernatant was removed, and the pellet was washed twice with deionized water to eliminate any unencapsulated drug [22].

Blank microspheres (PP-MS) were prepared using the same procedure. The final SIV-CAV-PP-MS formulations were stored in a refrigerator at 4 °C for stability before further evaluation. The optimized formulation was freeze-dried (VirTis Advantage, Warminster, PA, USA) according to the procedure described by Zhou Y et al. [23].

### 2.3. Characterisation of SIV-CAV-PP-MS Formulations

#### 2.3.1. Scanning Electron Microscopy (SEM)

Microspheres were mounted on carbon tape and sputter-coated with a thin layer of gold/palladium. The morphology of the microspheres was examined using a scanning electron microscope (JEOL SEM-5000, Tokyo, Japan). SEM images (*n* = 3) were analyzed with image analysis software to assess the morphology of the microspheres.

#### 2.3.2. The Size, Zeta Potential and Polydispersity Index (PDI) of Microspheres

Three parallel batches of SIV-CAV-PP-MS were dispersed in deionized water by sonication to measure their particle PDI. The size and zeta potential (ζ) of the SIV-CAV-PP-MS in deionized water was determined by simultaneously using the zeta potential analysis mode of the laser particle size analyzer and potential measuring devices (Malvern Instruments Co., Malvern, UK).

#### 2.3.3. Determination of SIV-CAV-PP-MS Entrapment Efficiency

The encapsulation efficiency of SIV and CAV was assessed using an Agilent C18 column. Drug concentrations were measured by high-performance liquid chromatography (HPLC) with a mobile phase consisting of sodium dihydrogen phosphate solution and acetonitrile (35:65, *v*/*v*) or acetonitrile and water (43:57, *v*/*v*) at a flow rate of 1.0 mL/min. The amount of SIV and CAV in the supernatants was quantified using HPLC at 238 nm and 280 nm absorbance wavelengths. The drug content in microspheres was m_1_, and the drug dosage was calculated as the total drug dose (*m*_2_). The encapsulation efficiency (EE) of the drug was calculated according to the following Formula (1)

(1) $$\mathrm{EE}\%=\frac{m_{1}}{m_{2}}\times 100\%$$

#### 2.3.4. Fourier Transform Infrared Spectroscopy (FTIR)

The material distribution within the pellets was analyzed using Fourier transform infrared spectroscopy combined with synchrotron radiation. FTIR spectra of SIV-CAV-PP-MS were obtained by placing powdered samples on a crystal for analysis at room temperature. FTIR spectra were recorded between 400 and 4000 cm^−1^ using a Spectrum100 FTIR Spectrometer (PerkinElmer, Waltham, MA, USA) [24].

#### 2.3.5. Differential Scanning Calorimetry (DSC)

To evaluate the compatibility of the ingredients, a DSC thermogram of SIV, blank PP-MS, the physical mixture, and SIV-CAV-PP-MS was obtained using a DSC analyzer system (Beijing Hengjiu DSC, Beijing, China). Samples were placed in hermetic pans and heated from 25.0 to 200.0 °C at 10 °C/min. Nitrogen gas was purged during measurement to maintain an inert atmosphere [25].

#### 2.3.6. Stability Studies

Stability studies were conducted on 10 mg of optimized freeze-dried SIV-CAV-PP-MS for one month at 40 °C ± 2 °C and 75% RH ± 5% RH. The morphology of the SIV-CAV-PP-MS was examined on the 1st and 30th days.

### 2.4. In Vitro Drug Release

In vitro drug release was evaluated using both direct release and ultracentrifugation methods. The solubility of SIV-CAV-PP microspheres (SIV-CAV-PP-MS) was assessed by dispersing the samples in screw-capped glass vials containing 5 mL of phosphate-buffered saline (PBS) with pH 7.4 and 0.5% sodium dodecyl sulfate (SDS). The vials were shaken in an incubator at 37 °C. A total of 5 mL of release medium was removed and replaced with fresh buffer, and the samples were collected daily for 30 days. Each sample was filtered through a 0.22 μm nylon membrane, and each experiment was done in triplicate. The concentrations of SIV and CAV in the resulting filtrate were quantified by high-performance liquid chromatography (HPLC) and reported as the amounts of SIV and CAV released from the system in soluble form [26]. Determination of the content of SIV and CAV in the release medium was assessed using an Agilent C18 column. Drug concentrations were measured by high-performance liquid chromatography (HPLC) with a mobile phase consisting of Glacial acetic acid solution 0.15% and acetonitrile (36:64, *v*/*v*) or acetonitrile and water (43:57, *v*/*v*) at a flow rate of 1.0 mL/min. Cumulative release (*Q*) per point was calculated according to Formula (2). (*Cn*) is the concentration of the drug at each sampling point; (*V*) is the sample volume; (*W*) and (*D*) are the mass and drug loading of the microspheres, respectively.

(2) $$Q\%=\frac{(C_{n}+C_{n-1}+C_{n-2}+...+C_{1})V}{W\times D}\times 100$$

### 2.5. In Vivo Release Kinetics

Thirteen male albino rats, each weighing between 80 and 100 g, were purchased from Changchun Yisi Experimental Animal Technology Co., Ltd. (Changchun, China). The rats were divided into two groups: given the constraints on injection volume associated with subcutaneous and intramuscular administration of microspheres, intraperitoneal injection was chosen as the method for administering SIV-CAV-PP-MS and SIV-CAV suspensions. The two groups of experimental rats were given same dosages of drugs (SIV 36 mg/kg and CAV 90 mg/kg). Group I (SIV CAV suspension group) received a drug suspension solution via intraperitoneal injection. In contrast, Group II (SIV-CAV-PP-MS group) received an intraperitoneal injection of SIV-CAV-PP-MS (1.6 g of SIV-CAV-PP-MS dissolved in normal saline). Prior to dosing, 2 mL of blood was collected from each rat. Post-dosing, approximately 1.5 mL of blood was collected at various time points (0.083, 0.25, 0.5, 0.75, 1, 2, 4, 8, 12 h for the suspension group and 0.083, 0.25, 0.5, 1, 2, 3, 4, 5, 7, 9, 12, 15, 18, 21 days for the SIV-CAV-PP-MS group) via retro-orbital bleeding under mild anaesthesia. The collected blood was centrifuged at 5000 rpm for 10 min, and the plasma (100 μL) was stored at −80 °C for further analysis [27].

For analysis, 100 μL of acetonitrile and 10 μL of an internal standard (lovastatin or methyl eugenol) were added to 100 μL of plasma to precipitate proteins. The mixture was vortexed and centrifuged at 10,000 rpm for 10 min. HPLC analyzed the supernatant, and pharmacokinetic parameters were determined using DAS 2.0. Statistical analysis was performed using an unpaired *t*-test, with *p* ≤ 0.01 considered statistically significant [28].

### 2.6. In Vivo Studies

Animals: Seventy-two male MK mice aged 2–8 weeks were obtained from Changchun Yisi Experimental Animal Technology Co., Ltd. The mice were acclimatized for one week and randomly assigned to nine groups (*n* = 8 each). Ethics Committee approval number: JDXYX-2024017.

#### 2.6.1. Lipid-Lowering Research

Six groups were used to evaluate lipid-lowering efficacy.

Hyperlipidemia model group (MC Group): received Poloxamer 407 (P407) (500 mg/kg/day) in distilled water via intraperitoneal injection twice a week for 4 weeks.

In order to prevent severe liver damage caused by high-fat intake, which could interfere with the evaluation of simvastatin-induced liver injury, hyperlipidemia in mice was induced by repeated intraperitoneal injections of P407 rather than by the conventional method of administering a high-fat emulsion or diet. This approach allowed for a simpler and faster model induction of persistent hyperlipidemia [29].

Positive drug control group (PC Group): received P407 for 4 weeks and were treated with alginic sodium (10.50 mg/kg/day) via intraperitoneal injection for 20 days, starting on the 11th day of dyslipidemia induction.

Simvastatin control group (SIV Group): received P407 for 4 weeks and were treated with Simvastatin suspension 10 mL/kg/day (SIV 0.9 mg/kg/d) via intraperitoneal injection for 20 days, starting on the 11th day of hyperlipidemia induction.

SIV-CAV-PP-MS Treatment Groups: Compromised of: SIV-CAV-PP-MS high-dose group (SC-H Group), SIV-CAV-PP-MS medium-dose group (SC-M Group) and SIV-CAV-PP-MS low-dose group (SC-L Group). Mice with hyperlipidemia received SIV-CAV-PP-MS 20, 10, or 5 mL/kg/20 days (SIV: 36.00, 18.00, 9.00 mg/kg/20 d) via a single-dose intraperitoneal injection, starting on the 11th day of hyperlipidemia induction. All animals were treated for 20 days before blood and tissue sample collection [30].

#### 2.6.2. Side Effect Mitigation Research

Three groups were used for assessing side effect mitigation:

Blank control group (NC Group): Served as the standard control group.

SIV side effects model group (SIVM Group): received P407 for 4 weeks and were treated with Simvastatin suspension 15 mg/kg/day via intraperitoneal injection for 20 days, starting on the 11th day of dyslipidemia induction [17].

SIV-CAV-PP-MS treatment group (SCMT Group): received P407 for 4 weeks and were treated with SIV-CAV-PP-MS 20 mL/kg/20 days (SIV 300 mg/kg/20 d) via a single-dose intraperitoneal injection, starting on the 11th day of hyperlipidemia induction.

#### 2.6.3. Histopathologic Examination

After 20 days of treatment, blood and tissue samples were collected. Blood was drawn from the inner canthus of the eye, centrifuged at 3000 rpm for 15 min to collect sera, and stored at −80 °C for biochemical analyses. Mice were euthanized by cervical dislocation, and the liver and gastrocnemius muscles were excised, washed with cold saline, and preserved in neutral buffered formalin (10%) for histopathological examination. Liver sections were stained with Hematoxylin and Eosin (H&E) and Oil Red O, while skeletal muscle sections were stained with H&E, and both were examined under a light microscope.

#### 2.6.4. Behavioral Testing of Exercise Tolerance

Locomotor endurance was assessed in the NC, SIVM, and SCMT groups before modelling and at the end of the experiment [17]. Front Leg Tensile Test: Mice were positioned on a grid basket by their forepaws, and a dynamometer was attached to their tails. The force was measured by pulling the dynamometer, and the test was repeated three times with a one-hour interval between tests. Grid Flipping Suspension Test: Mice were placed on a grid basket and rotated clockwise at 45 degrees per second. The hanging time was recorded, and the test was repeated three times with a one-hour interval between tests.

#### 2.6.5. Biochemical Analysis

Total cholesterol (TC), triglycerides (TG), high-density lipoprotein cholesterol (HDL-C), and low-density lipoprotein cholesterol (LDL-C) levels in serum samples were measured using colourimetric kits (Nanjing Jiancheng Technology Co., Nanjing, China) and a microplate reader (Tecan GmbH 5802, Grödig, Austria) according to the manufacturer’s instructions. Results were expressed as mg/dL.

#### 2.6.6. Statistical Analysis

Data are presented as mean ± standard deviation. Statistical differences between means were assessed using ANOVA with Prism software (version 8.0; GraphPad, San Diego, CA, USA). A *p*-value of ≤ 0.05 was considered statistically significant. Significance was inferred at * *p* < 0.05, ** *p* < 0.01, and *** *p* < 0.001.

## 3. Results and Discussion

### 3.1. Morphology of SIV-CAV-PP-MS

**Figure 2:**
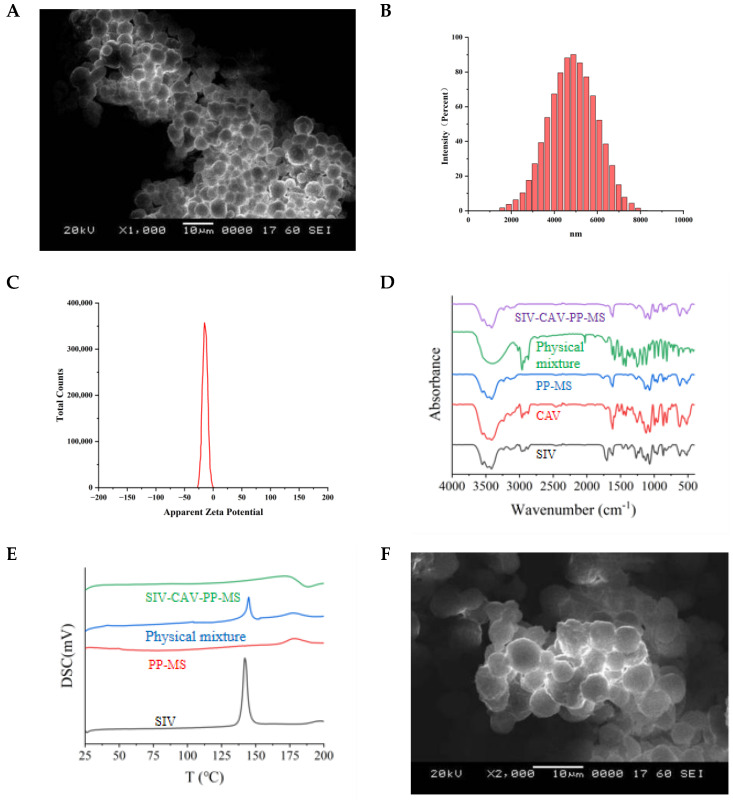

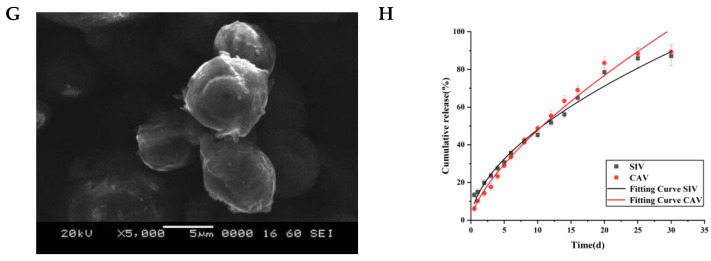
SEM image of SIV-CAV-PP-MS (**A**). Particle size distribution of SIV-CAV-PP-MS (**B**). Zeta potential of SIV-CAV-PP-MS (**C**). Fourier-transform infrared spectroanalysis (**D**). Differential scanning for thermal analysis (**E**). SEM of the SIV-CAV-PP-MS ((**F**): 1st day, (**G**): 30th day) (**F**,**G**). In vitro release fitting curves of SIV-CAV-PP-MS in PBS (pH 7.4) with 0.5% SDS (**H**).

The surface morphology of SIV-CAV-PP-MS was analyzed using scanning electron microscopy (SEM), as shown in Figure 2A. The microspheres exhibited a uniform and smooth spherical shape with an average size of 4 μm. The microspheres, which ranged in size from 1 to 15 μm, can be phagocytosed by reticuloendothelial system macrophages, making them suitable for general injection [31,32]. Microspheres with this size range meet the injection requirements.

### 3.2. The Size, Zeta Potential and Polydispersity Index (PDI) of Microspheres

The average particle diameter was 4.25 ± 2.53 μm (Figure 2B), with results similar to those demonstrated in Figure 2A. The zeta potential, which describes the surface charge of the particles and reflects the degree of repulsion between charged particles in a dispersion, was measured at −17.01 mV, as shown in Figure 2C. The polydispersity index (PDI) was 0.211 ± 0.078, indicating good dispersibility and the ability to pass through capillaries without causing blockages. The PDI indicates the uniformity of particle size distribution, with values below 0.1 representing monodispersity [33].

### 3.3. Fourier-Transform Infrared Spectroscopy (FTIR) Analysis

Figure 2D shows SIV peaks at 1268.04, 1722.67, and 2952.97 cm^−1^. CAV peaks at 1618.37, 1390.66, 1385.52 and 1375.17 cm^−1^. For the blank PEG-PLGA microspheres (PP-MS), the C=O stretching band appears at 1745.61 cm^−1^, and the characteristic ester absorption peaks of PLGA are found within the 1330–1050 cm^−1^ range. In the physical mixture, additional peaks at 1268.04, 1385.52, 1618.37, 1722.67, and 2952.97 cm^−1^ correspond to the drug and the support material. A comparison of the FTIR spectra in the 4000–500 cm^−1^ region between the PP-MS and the SIV-CAV-PP-MS shows no significant interaction, indicating that the drug was successfully encapsulated within the microspheres.

### 3.4. Differential Scanning Calorimetry (DSC)

DSC is a thermoanalytical technique that measures the differences in heat required to maintain a sample and a reference at the same temperature. Figure 2E presents the thermograms for SIV, blank PEG-PLGA microspheres (PP-MS), the physical mixture, and SIV-CAV-PP-MS. The DSC thermogram of SIV shows a melting endothermic peak at 144.06 °C. For blank PP-MS, the melting endothermic peak occurs at 179.21 °C. In the physical mixture, the melting endothermic peaks of both SIV and blank PP-MS are present but with reduced peak intensities. Additionally, the thermogram of SIV-CAV-PP-MS demonstrates either the disappearance or significant flattening of the SIV melting peak, indicating the encapsulation effect of the inclusion complexes.

### 3.5. Stability Study

The stability data for the microspheres indicate significant changes in flowability and morphology over the one-month accelerated stability test period (Figure 2F). At the end of the month, pronounced aggregation and deformation were observed, suggesting a potential decrease in microsphere stability under accelerated conditions. As depicted in Figure 2G, the polymeric microparticles tend to adhere to one another and aggregate. Additionally, the particle size of the SIV-CAV-PP-MS microspheres changed over one month. With the increase of time from the 1st to the 30th day, the size of the microspheres exhibited a slight increase. Excessively high temperatures may lead to the loosening and deformation of the PEG-PLGA polymeric scaffold. As such, the adhesion and deformation may be attributed to the poor stability of PEG-PLGA under high temperature and high humidity conditions.

### 3.6. In Vitro Release Studies

Table 1 presents the solubility data of SIV and CAV in various release media containing surfactants. Based on these results, pH 7.4 PBS with 0.5% SDS was selected as the release medium.

The dissolution profiles of SIV-CAV-PP-MS in pH 7.4 PBS containing 0.5% SDS are shown in Figure 2G,H. The release pattern of SIV-CAV-PP-MS exhibited a slight burst release at 0.5 days. The cumulative drug release increased with higher SIV and CAV levels in the release medium, reaching 88.91% and 89.35% at 25 days, respectively. The drug-loaded SIV-CAV-PP-MS demonstrated a slow and sustained release profile for both drugs, with high cumulative release rates.

It is well known that the initial drug burst from the PEG-PLGA microsphere results from the fast hydrolytic degradation of PLGA in forming pores large enough for the diffusion of drug molecules. The PVA at the core and surface of the microspheres could minimize the initial drug burst typically found with microspheres. In this study, the slight burst is likely due to drug molecules physically adsorbed on the particle surface, which diffuses first into the release medium. Sampling at later time points showed that the amount of drug released was not constant but decreased, presumably caused by irreversible adsorption on the polymer surface. Previous in vitro studies suggest that drug diffusion from microspheres slows in later stages due to gel formation [34]. Similar findings have been reported, such as Quercetin sustained-release microspheres showing 80.84% release over 24 days [35]. One objective of controlled drug delivery systems is to achieve sustained drug release over extended periods.

To investigate the mechanism of release from SIV-CAV-PP-MS, in vitro release data were fitted to zero-order, first-order, Higuchi, and Ritger–Peppas models. These four models are commonly utilized to delineate the mechanism by which a drug is released from a carrier system [36]. The fitted equations are presented in Table 2. The linear relationship coefficients after fitting the dialysis model are as follows: Ritger–Peppas >Zero-order > First-order/Higuchi. The Higuchi and Ritger–Peppas equations are frequently utilized to describe the release kinetics of a specific solute during diffusion processes occurring on the surface of a colloidal or solid adsorbent material. These equations are pertinent to research endeavors within the domain of solid pharmaceutical formulations. Based on the fitting of the Ritger–Peppas model, the release mechanism of drugs within sustained-release matrices was investigated [37].

The Ritger–Peppas model provided the best fit, with correlation coefficients of r_SIV_ = 0.9886 and r_CAV_ = 0.9889, indicating its suitability for describing the release kinetics. In the Ritger–Peppas model, the release data in pH 7.4 PBS with 0.5% SDS, where cumulative drug release exceeded 85%, showed exponents n of 0.62 for SIV and 0.77 for CAV. These values, falling within the range of 0.45 to 0.89. The SIV-CAV-PP-MS release process of the Ritger–Peppas model changed from drug diffusion to skeleton erosion, and ultimately to a Fickian diffusion mechanism [26,38].

All results are shown as mean ± SD from a triplicated experiment (n = 3). Origin^®^ software version 2019 for Windows was used for the statistical analysis.

### 3.7. Bioavailability Study

**Figure 3:**
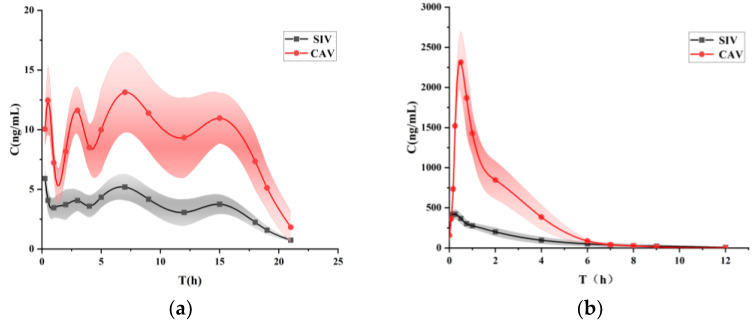
Plasma concentration–time curves in rats following injection of SIV-CAV-PP-MS (**a**). Plasma concentration–time curves in rats following injection of SIV-CAV suspension (**b**). Note: the translucent area is the error band of the graph.

The in vivo pharmacokinetics of the suspension and SIV-CAV-PP-MS (intraperitoneal injection) were analyzed, with the blood plasma concentration–time profiles in Figure 3. In the SIV-CAV-PP-MS group, the concentrations of SIV and CAV reached their maximum levels at approximately seven days, whereas the suspension group exhibited peak concentrations around 0.25 h. This indicates that using PEG-PLGA as a carrier significantly delays drug release.

Noncompartmental pharmacokinetic parameters such as *C_max_*, *T_max_*, elimination rate constant, *AUC_total_*, *AUMC_total_*, and mean residence time (MRT) were calculated using DAS 2.0 software (Table 3). The MRT of the SIV-CAV-PP-MS formulation was significantly extended compared to the suspension, with values increasing from 2.45 ± 0.15 and 2.17 ± 0.25 h (for SIV and CAV, respectively) to 191.42 ± 12.78 and 211.86 ± 10.09 h (for SIV and CAV, respectively). This suggests a prolonged residence time at the absorption site. The SIV-CAV-PP-MS formulation demonstrated a 55.67-fold and 156.33-fold reduction in *C_max_* (for SIV and CAV, respectively), and the half-life (T_1/2_) increased by 110.42-fold and 165-fold (for SIV and CAV, respectively) compared to the suspension. The significantly extended half-life in the SIV-CAV-PP-MS group indicates that PEG modification substantially prolongs the drug’s circulation time. These results indicate that a more significant proportion of the drug from the microspheres is absorbed and utilized, demonstrating that the microsphere formulation enhances the bioavailability of the drug in hyperlipidemic rats.

The enhanced bioavailability of SIV and CAV in the SIV-CAV-PP-MS formulation can be attributed to the extended-release time of the microspheres due to PEG-PLGA, which increases the residence time and controlled release of the drug. Consequently, this formulation mitigates issues related to rapid drug release and bioavailability barriers. Parameters such as *T*_1/2_, *AUC*, and *MRT_last_* were significantly improved in the SIV-CAV-PP-MS group compared to the suspension group. These results confirm that encapsulating the drug in PEG-PLGA microspheres markedly extends its circulation time in vivo.

### 3.8. In Vivo Evaluation

#### 3.8.1. Investigation of the Lipid-Lowering Effects of SIV-CAV-PP-MS

##### The Body Weight and the Levels of TC, TG, HDL-C, and LDL-C Detection

**Figure 4:**
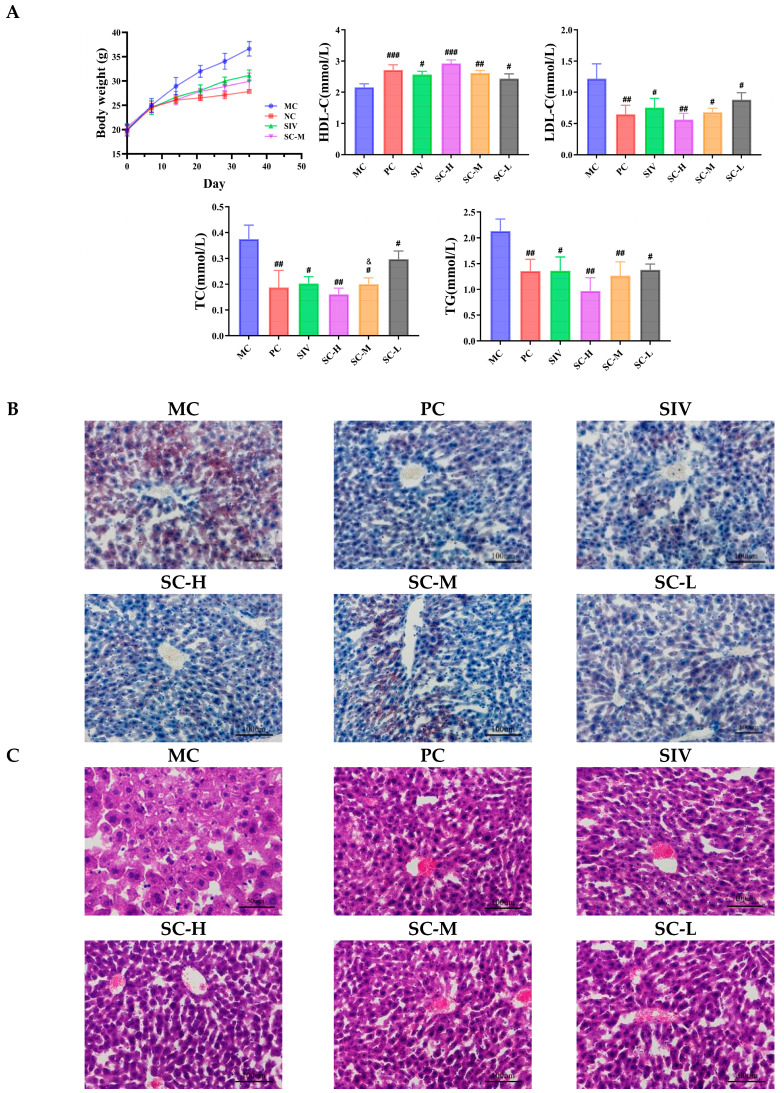
Effects of SIV-CAV-PP-MS on blood lipid levels. Compared with MC, ^#^ *p* < 0.05, ^##^ *p* < 0.01, ^###^ *p* < 0.01; Compared with SIV, ^&^ *p* < 0.05 (**A**). Oil red O staining of hepatic tissue of mice (**B**) and H&E staining of hepatic tissue of mice (**C**).

In Figure 4A, mice fed with SIV and SIV-CAV-PP-MS exhibited significantly reduced final body weight (SIV: 13.84%, SC-M: 17.63%) compared with the values obtained from the MC group mice. However, the importance of the analysis of body weight is not significant. In P407-treated mice, inflammatory cytokines, which increase in a hyperlipidemic state, may act directly on fat cells to increase leptin secretion without affecting body weight gain [39,40]. Each treatment group exhibited a significant decrease (*p* < 0.01) in blood lipid levels following a single treatment. Notably, the treated mice showed marked reductions in total cholesterol (TC), triglycerides (TG), and low-density lipoprotein cholesterol (LDL-C). In contrast high-density lipoprotein cholesterol (HDL-C) levels were significantly elevated compared to the MC group. Specifically, the SC-M group significantly reduced TC levels compared to the SIV group (*p* < 0.05). The TC levels were significantly reduced by 0.23-fold and 0.25-fold in the SIV and SC-M groups, respectively (*p* < 0.05). The addition of CAV significantly enhanced the lipid-lowering effects of SIV. The existing study suggests that CAV appears to potentially inhibit visceral adipogenesis and attenuate the production of pro-inflammatory cytokines in the visceral adipose [13]. CAV can potentiate the hypolipidemic effect of SIV. Both SIV and CAV alleviated the oxidative stress induced by P407 [14].

##### Histological Examinations

The liver plays a crucial role in lipid metabolism. Oil Red O staining revealed significant lipid droplet accumulation in the MC group, whereas SIV-CAV-PP-MS treatment notably reduced P407-induced hepatic fat deposition (Figure 4B). H&E staining showed that the MC group had enlarged hepatocytes, disordered hepatic cords, numerous fat vacuoles, lightly stained cytoplasm, and congestion in the portal veins and sinusoids (Figure 4C). In contrast, liver tissues from the SC-M group and those treated with SIV-CAV-PP-MS exhibited a significant reduction in vacuolar hepatic lipid accumulation, with minimal inflammatory cell infiltration in the portal area. SIV-CAV-PP-MS treatment notably improved liver histology.

Post-treatment with SIV or SIV-CAV-PP-MS substantially mitigated abnormal lipid accumulation in the liver. Specifically, compared to the SIV group, the SC-M group displayed histological features more closely resembling those of normal mice, with a well-organized hepatocyte structure, the greatest reduction in lipids, and overall improved liver health.

#### 3.8.2. Investigation of the Adverse Effects Mitigation of SIV-CAV-PP-MS

##### Behavioral Parameters

**Figure 5:**
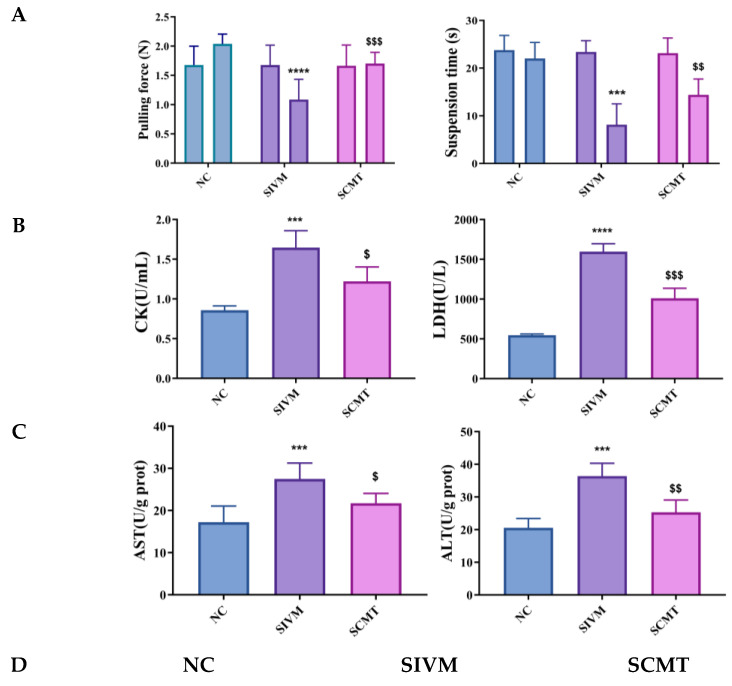

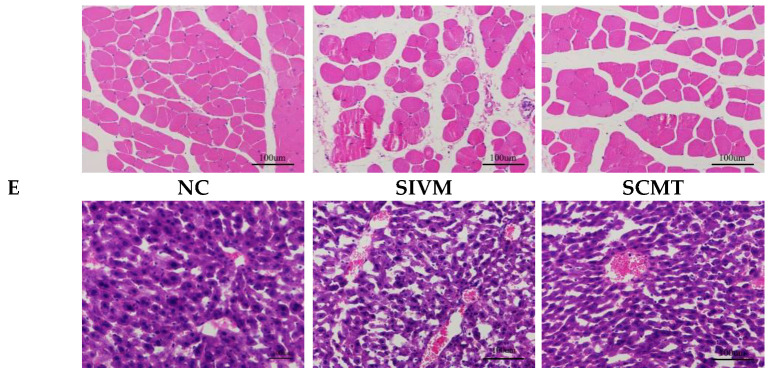
Assessment of exercise capacity in mice (**A**). The levels of CK and LDH detection (**B**). The Levels of AST and ALT detection (**C**). H&E staining of muscle tissue of mice (**D**) and H&E staining of hepatic tissue of mice (**E**). Compared with NC, *** *p* < 0.001, **** *p* < 0.0001; compared with SIVM, ^$^ *p* < 0.05, ^$$^ *p* < 0.01, ^$$$^ *p* < 0.001.

Reduced locomotor ability is a prominent feature of statin-induced myotoxicity [17]. Figure 5A illustrates the results of the forepaw grip test and the grid flip test. Initially, the performance of all groups was similar, with no significant differences observed. However, in the second test conducted before euthanasia, the forepaw grip strength of the SIVM group was significantly lower compared to the NC group. Conversely, the grip strength of the SCMT group was notably higher than that of the SIVM group. A previous study showed that SIV resulted in decreased improvements in exercise capacity in ApoE^−/−^ mice [8]. Accordingly, we took advantage of this model for our present study. Exercise tolerance intuitively corresponds to exercise performance. These differences were statistically significant, confirming the SIV injury model’s validity and demonstrating SIV-CAV-PP-MS’s efficacy in ameliorating muscle injury.

##### The Levels of CK, LDH, AST and ALT Detection

Creatine kinase (CK) and lactate dehydrogenase (LDH) are commonly used biomarkers for assessing skeletal muscle injury. Figure 5B display the serum levels of CK and LDH in the different mouse groups. Compared to the NC group, the SIVM group exhibited significantly elevated levels of serum CK (*p* < 0.001) and LDH (*p* < 0.0001). These findings confirm that the SIV injury model induces substantial muscle damage, consistent with the previous literature [39]. In contrast, treatment with SIV-CAV-PP-MS resulted in a reduction of CK and LDH levels by 0.17-fold (*p* < 0.05) and 0.37-fold (*p* < 0.001), respectively, compared to the SIVM group. In a previous study, elevated plasma CK and lowered plasma LDH were reported to be manifestations of SIV-induced skeletal muscle injury [41]. SIV-CAV-PP-MS alleviated the increase in the plasma level of CK and reduced the plasma level of LDH, which is induced by statins. These results indicate that SIV-CAV-PP-MS mitigated SIV-induced mitochondrial dysfunction.

Figure 5C presents the serum levels of AST and ALT in mice. Injection of SIV significantly elevated both AST and ALT levels compared to the standard control group. The SIVM group exhibited a significant change (*p* < 0.001) in AST and ALT levels compared to the NC group. Notably, the elevated serum concentrations of AST and ALT observed in the SIV-treated group were attenuated by the combination of CAV. Severe liver damage in the SIV group was consistent with findings reported in the literature [42]. The SCMT group showed a significant reduction in AST (0.21-fold decrease, *p* < 0.05) and ALT (0.30-fold decrease, *p* < 0.01) levels compared to the SIVM group. These results indicate that the encapsulation of CAV into MS effectively mitigates the hepatotoxicity induced by SIV.

##### Histological Examinations

To confirm the protective effect of SIV-CAV-PP-MS on the muscles and liver, we investigated the change in histomorphology using H&E staining. Histological examination of the quadriceps muscles from the NC group revealed normal muscle structure. Figure 5D shows muscle sections from normal control mice exhibited a typical histological appearance of muscle fibres. In contrast, histopathological analysis of the skeletal muscle from the SIVM group revealed extensive degeneration of muscle fibres, lymphocyte infiltration, and interstitial haemorrhaging. Mice treated with SIV-CAV-PP-MS displayed minimal inflammation, with muscle fibres showing some replacement by fibrous tissue in certain areas. Importantly, treatment with SIV-CAV-PP-MS led to a normalization of muscle tissue histology. Hematoxylin and eosin (H&E) staining of the liver demonstrated that the NC group had normal hepatic lobules, orderly arranged hepatic cords around the central vein, and visible hepatic sinusoids (Figure 5E). Conversely, SIVM-treated hyperlipidemic mice showed degenerative changes, including vacuolar degeneration of hepatocytes and inflammatory cell infiltration. Treatment with SIV-CAV-PP-MS improved liver histology, with the liver exhibiting close to normal histological appearance.

## 4. Conclusions

PEG-PLGA-based biodegradable microspheres have been widely used in drug delivery in the past decades. In this study, we successfully prepared dual-loaded Long-Circulating microspheres with uniform particle size, and good biocompatibility using the solvent evaporation method. The particle size satisfied the injection requirements. The in vitro sustained release effect was good, and showed longer circulation time than the free drug. SIV-CAV-PP-MS, as a long-acting sustained-release formulation, controlled the drug release rate, reduced the peak concentration of the drug in vivo, improved drug safety, and improved the bioavailability of the drug in rats.

Our findings demonstrate that SIV-CAV-PP-MS effectively regulates hyperlipidemia and improves dysfunctional hepatic lipid metabolism in P407-induced hyperlipidemic mice. Furthermore, the combined application of Simvastatin and Carvacrol overcame the side effects of Simvastatin, and effectively enhanced lipid-lowering efficacy, achieving a synergistic lipid-lowering effect where 1 + 1 > 2. Overall, SIV-CAV-PP-MS would be a promising delivery system for the treatment of hyperlipidemia, demonstrating a strong potential for clinical transformation.

## Figures and Tables

**Table 1:** Saturation solubility of SIV and CAV in various dissolution media.

	Solvents	Solubility (mg/L)
SIV	PBS (pH7.4) + 0.5% Tween-80	315.57
	PBS (pH7.4) + 0.5% SDS	346.17
CAV	PBS (pH7.4) + 0.5% Tween-80	6830.00
	PBS (pH7.4) + 0.5% SDS	6230.00

**Table 2:** The release patterns equations of SIV-CAV-PP-MS.

	Model	Equation	r
SIV	Zero-order kinetics	Mt = 3.32k + 11.82	0.9775
	First-order kinetics	Mt = 104.69(1 − e^−0.07^)	0.9043
	Higuchi equation	Mt = 17.33x^1/2^ − 4.43	0.9642
	Ritger–Peppas equation	Qt = 11.69x^0.62^	0.9886
CAV	Zero-order kinetics	Mt = 5.61k + 3.94	0.9747
	First-order kinetics	Mt = 143.45(1 − e^−0.04^)	0.9807
	Higuchi equation	Mt = 20.45x^1/2^ − 13.45	0.9654
	Ritger–Peppas equation	Qt = 8.07x^0.77^	0.9889

**Table 3:** Pharmacokinetic parameters of SIV-CAV-PP-MS and SIV-CAV suspensions following administration.

Parameter	SIV-CAV-PP-MS		SIV-CAV-Suspensions	
	SIV	CAV	SIV	CAV
*t*_1/2_ (h)	206.48 ± 55.07 *	156.75 ± 48.66 *	1.87 ± 0.12	0.95 ± 0.06
*T_max_* (h)	87 ± 93.53 *	100 ± 94.80 *	0.25 ± 0.00	0.5 ± 0.00
*C_max_* (ng/mL)	6.302 ± 1.313 *	15.01 ± 1.29 *	350.71 ± 42.57	2346.52 ± 231.05
*AUC_last_* (h ng/mL)	1745.23 ± 195.81	5120.41 ± 518.27	1213.97 ± 106.35	4631.42 ± 753.51
*AUC_inf_* (h ng/mL)	1875.54 ± 293.86	5327.48 ± 655.86	1228.72 ± 112.97	4647.68 ± 749.42
*MRT_last_* (h)	191.42 ± 12.78 *	211.86 ± 10.09 *	2.45 ± 0.15	2.17 ± 0.25

Note: Compared with SIV-CAV-suspensions * *p* < 0.01.

## Data Availability

Data for this article, including pictures and forms, are available at [Science Data Bank] at [DOI:10.57760/sciencedb.15181].

## References

[1] Abdel-Maksoud M., Sazonov V., Gutkin S.W., Hokanson J.E. (2008). Effects of modifying triglycerides and triglyceride-rich lipoproteins on cardiovascular outcomes. J. Cardiovasc. Pharmacol..

[2] Li H., Rabearivony A., Zhang W., Chen S., An X., Liu C. (2020). Chronopharmacology of simvastatin on hyperlipidaemia in high-fat diet-fed obese mice. J. Cell. Mol. Med..

[3] Ding L., Chen C., Yang Y., Fang J., Cao L., Liu Y. (2022). Musculoskeletal Adverse Events Associated with PCSK9 Inhibitors: Disproportionality Analysis of the FDA Adverse Event Reporting System. Cardiovasc. Ther..

[4] Stewart J., McCallin T., Martinez J., Chacko S., Yusuf S. (2020). *Hyperlipidemia*. Pediatr. Rev..

[5] Hirota T., Fujita Y., Ieiri I. (2020). An updated review of pharmacokinetic drug interactions and pharmacogenetics of statins. Expert Opin. Drug Metabolsim Toxicol..

[6] Backes J.M., A Howard P., Ruisinger J.F., Moriarty P.M. (2009). Does simvastatin cause more myotoxicity compared with other statins?. Ann. Pharmacother..

[7] Ali A.U., Abd-Elkareem M., Kamel A.A., Abou Khalil N.S., Hamad D., Nasr N.E.H., Hassan M.A., El Faham T.H. (2023). Impact of porous microsponges in minimizing myotoxic side effects of simvastatin. Sci. Rep..

[8] Jiang B., Yang Y.J., Dang W.Z., Li H., Feng G.Z., Yu X.C., Shen X.Y., Hu X.G. (2020). Astragaloside IV reverses simvastatin-induced skeletal muscle injury by activating the AMPK-PGC-1α signalling pathway. Phytother. Res..

[9] González-Iglesias E., Ochoa D., Navares-Gómez M., Zubiaur P., Aldama M., de la Torre T., Ríos-Rodríguez M.L., Soria-Chacartegui P., Rodríguez-Lopez A., Abad-Santos F. (2024). Evaluation of the role of metabolizing enzymes and transporter variants in ezetimibe pharmacokinetics. Front. Pharmacol..

[10] Ahmed Shaikh N., Khakid S., Alam M., Hasan S.S., Rizvi F., Mobeen K. (2024). Comparative and combination study of simvastatin alone and in combination with Beta vulgaris in hyperlipidemia patients. Pak. J. Pharm. Sci..

[11] Gholijani N., Abolmaali S.-S., Kalantar K., Ravanrooy M.-H. (2020). Therapeutic Effect of Carvacrol-loaded Albumin Nanoparticles on Arthritic Rats. Iran. J. Pharm. Res..

[12] Khalil M., Serale N., Diab F., Baldini F., Portincasa P., Lupidi G., Vergani L. (2022). Beneficial Effects of Carvacrol on In Vitro Models of Metabolically-Associated Liver Steatosis and Endothelial Dysfunction: A Role for Fatty Acids in Interfering with Carvacrol Binding to Serum Albumin. Curr. Med. Chem..

[13] Cho S., Choi Y., Park S., Park T. (2012). Carvacrol prevents diet-induced obesity by modulating gene expressions involved in adipogenesis and inflammation in mice fed with high-fat diet. J. Nutr. Biochem..

[14] El Aal H.A.A., Ahmed L.A., Hassan W.A., Fawzy H.M., Moawad H. (2017). Combination of carvacrol with simvastatin improves the lipid-lowering efficacy and alleviates simvastatin side effects. J. Biochem. Mol. Toxicol..

[15] Ellison D.K., Moore W.D. (1999). Simvastatin. Analytical Profiles of Drug Substances and Excipients.

[16] Kelley W.J., Fromen C.A., Lopez-Cazares G., Eniola-Adefeso O. (2018). PEGylation of model drug carriers enhances phagocytosis by primary human neutrophils. Acta Biomater..

[17] Alavi M., Webster T.J. (2021). Recent progress and challenges for polymeric microsphere compared to nanosphere drug release systems: Is there a real difference?. Bioorganic Med. Chem..

[18] Jusu S.M., Obayemi J.D., Salifu A.A., Nwazojie C.C., Uzonwanne V., Odusanya O.S., Soboyejo W.O. (2020). Drug-encapsulated blend of PLGA-PEG microspheres: In vitro and in vivo study of the effects of localized/targeted drug delivery on the treatment of triple-negative breast cancer. Sci. Rep..

[19] Anwar A., Sun P., Rong X., Arkin A., Elham A., Yalkun Z., Li X., Iminjan M. (2023). Process analytical technology as in-process control tool in semi-continuous manufacturing of PLGA/PEG-PLGA microspheres. Heliyon.

[20] Wang P., Zhuo X., Chu W., Tang X. (2017). Exenatide-loaded microsphere/thermosensitive hydrogel long-acting delivery system with high drug bioactivity. Int. J. Pharm..

[21] Buske J., König C., Bassarab S., Lamprecht A., Mühlau S., Wagner K.G. (2012). Influence of PEG in PEG-PLGA microspheres on particle properties and protein release. Eur. J. Pharm. Biopharm..

[22] Andhariya J.V., Burgess D.J. (2016). Recent advances in testing of microsphere drug delivery systems. Expert Opin. Drug Deliv..

[23] Zhou Y., Liu B. (2019). Preparation of chitosan microcarriers by high voltage electrostatic field and freeze drying. J. Biosci. Bioeng..

[24] Hakiem A.F.A., Mohamed N.A., Ali H.R. (2021). FTIR spectroscopic study of two isostructural statins: Simvastatin and Lovastatin as authentic and in pharmaceuticals. Spectrochim. Acta Part A Mol. Biomol. Spectrosc..

[25] Suriyaamporn P., Sahatsapan N., Patrojanasophon P., Opanasopit P., Kumpugdee-Vollrath M., Ngawhirunpat T. (2023). Optimization of In Situ Gel-Forming Chlorhexidine-Encapsulated Polymeric Nanoparticles Using Design of Experiment for Periodontitis. AAPS PharmSciTech.

[26] Narita A., Nakano Y., Okada H., Yamamoto T., Matsunaga N., Ikeda S., Izumi Y., Kitagawa A., Ota T., Suzuki K. (2023). In Vitro Characterization of Drug-Loaded Superabsorbent Polymer Microspheres: Absorption and Release Capacity of Contrast Material, Antibiotics and Analgesics. Cardiovasc. Interv. Radiol..

[27] Chen Z., Liu Z., Wang S., Cheng C., Sun X., Liu Z., Wei J., Jiang J., Lan H., Zhou M. (2023). Long-Circulating Lipid Nanospheres Loaded with Flurbiprofen Axetil for Targeted Rheumatoid Arthritis Treatment. Int. J. Nanomed..

[28] Gao S., Tian B., Han J., Zhang J., Shi Y., Lv Q., Li K. (2019). Enhanced transdermal delivery of lornoxicam by nanostructured lipid carrier gels modified with polyarginine peptide for treatment of carrageenan-induced rat paw edema. Int. J. Nanomed..

[29] Cheng D., Zhang M., Zheng Y., Wang M., Gao Y., Wang X., Liu X., Lv W., Zeng X., Belosludtsev K.N. (2024). α-Ketoglutarate prevents hyperlipidemia-induced fatty liver mitochondrial dysfunction and oxidative stress by activating the AMPK-pgc-1α/Nrf2 pathway. Redox Biol..

[30] Irwin J.C., Fenning A.S., Ryan K.R., Vella R.K. (2018). Validation of a clinically-relevant rodent model of statin-associated muscle symptoms for use in pharmacological studies. Toxicol. Appl. Pharmacol..

[31] Li Y., Liu S., Zhang J., Wang Y., Lu H., Zhang Y., Song G., Niu F., Shen Y., Midgley A.C. (2024). Elastic porous microspheres/extracellular matrix hydrogel injectable composites releasing dual bio-factors enable tissue regeneration. Nat. Commun..

[32] Zhang C., Yang L., Wan F., Bera H., Cun D., Rantanen J., Yang M. (2020). Quality by design thinking in the development of long-acting injectable PLGA/PLA-based microspheres for peptide and protein drug delivery. Int. J. Pharm..

[33] Harisa G.I., Alomrani A.H., Badran M.M. (2017). Simvastatin-loaded nanostructured lipid carriers attenuate the atherogenic risk of erythrocytes in hyperlipidemic rats. Eur.J. Pharm. Sci..

[34] Li K. (2020). Preparation, Evaluation, and Pharmacodynamic Study of Long-Acting Sustained-Release Microspheres of Dopamine Receptor Agonists.

[35] Zhang S., Zhou X., Qin Q., Na Q.I. (2021). Process Optimization, Characterization, and In Vitro Release Study of Quercetin Sustained-Release Microspheres Prepared by SPG Membrane Emulsification Method. Her. Med..

[36] Su Y., Liu J., Tan S., Liu W., Wang R., Chen C. (2022). PLGA sustained-release microspheres loaded with an insoluble small-molecule drug: Microfluidic-based preparation, optimization, characterization, and evaluation in vitro and in vivo. Drug Deliv..

[37] Wang M., Kong X.P., Li H., Ge J.C., Han X.Z., Liu J.H., Yu S.L., Li W., Li D.L., Wang J. (2023). Coprecipitation-based synchronous chlorantraniliprole encapsulation with chitosan: Carrier-pesticide interactions and release behavior. Pest. Manag. Sci..

[38] Wu B., Wu L., He Y., Yin Z., Deng L. (2021). Engineered PLGA microspheres for extended release of brexpiprazole: In vitro and in vivo studies. Drug Dev. Ind. Pharm..

[39] Yeom M., Park J., Lee B., Lee H.S., Park H.J., Won R., Lee H., Hahm D.H. (2018). Electroacupuncture ameliorates poloxamer 407-induced hyperlipidemia through suppressing hepatic SREBP-2 expression in rats. Life Sci..

[40] Liu Q., Liu S.N., Li L.Y., Chen Z.Y., Lei L., Zhang N., Shen Z.F. (2011). A dyslipidemia animal model induced by poloxamer 407 in golden hamsters and pilot study on the mechanism. Acta Pharm. Sin..

[41] Wei J., Huan Y., Heng Z., Zhao C., Jia L., Yu Y., Gao Y. (2022). Dynamic urine proteome changes in a rat model of simvastatin-induced skeletal muscle injury. J. Proteom..

[42] Hareedy M.S., Ahmed E.A., Ali M.F. (2019). Montelukast modifies simvastatin-induced myopathy and hepatotoxicity. Drug Dev. Res..

